# Role of Ions in the Regulation of Light-Harvesting

**DOI:** 10.3389/fpls.2016.01849

**Published:** 2016-12-16

**Authors:** Radek Kaňa, Govindjee

**Affiliations:** ^1^Institute of Microbiology, Academy of Sciences of the CzechiaTřeboň, Czechia; ^2^Faculty of Science, Institute of Chemistry and Biochemistry, University of South BohemiaČeské Budějovice, Czechia; ^3^Center of Biophysics and Quantitative Biology, Department of Biochemistry, Department of Plant Biology, University of Illinois at Urbana-ChampaignUrbana, IL, USA

**Keywords:** ions, non-photochemical quenching, state transitions, light-harvesting protein complexes, photosynthesis, photoprotection

## Abstract

Regulation of photosynthetic light harvesting in the thylakoids is one of the major key factors affecting the efficiency of photosynthesis. Thylakoid membrane is negatively charged and influences both the structure and the function of the primarily photosynthetic reactions through its electrical double layer (EDL). Further, there is a heterogeneous organization of soluble ions (K^+^, Mg^2+^, Cl^−^) attached to the thylakoid membrane that, together with fixed charges (negatively charged amino acids, lipids), provides an electrical field. The EDL is affected by the valence of the ions and interferes with the regulation of “state transitions,” protein interactions, and excitation energy “spillover” from Photosystem II to Photosystem I. These effects are reflected in changes in the intensity of chlorophyll *a* fluorescence, which is also a measure of photoprotective non-photochemical quenching (NPQ) of the excited state of chlorophyll *a*. A triggering of NPQ proceeds via lumen acidification that is coupled to the export of positive counter-ions (Mg^2+^, K^+^) to the stroma or/and negative ions (e.g., Cl^−^) into the lumen. The effect of protons and anions in the lumen and of the cations (Mg^2+^, K^+^) in the stroma are, thus, functionally tightly interconnected. In this review, we discuss the consequences of the model of EDL, proposed by Barber (1980b) Biochim Biophys Acta **594**:253–308) in light of light-harvesting regulation. Further, we explain differences between electrostatic screening and neutralization, and we emphasize the opposite effect of monovalent (K^+^) and divalent (Mg^2+^) ions on light-harvesting and on “screening” of the negative charges on the thylakoid membrane; this effect needs to be incorporated in all future models of photosynthetic regulation by ion channels and transporters.

## Introduction

**Figure 1:**
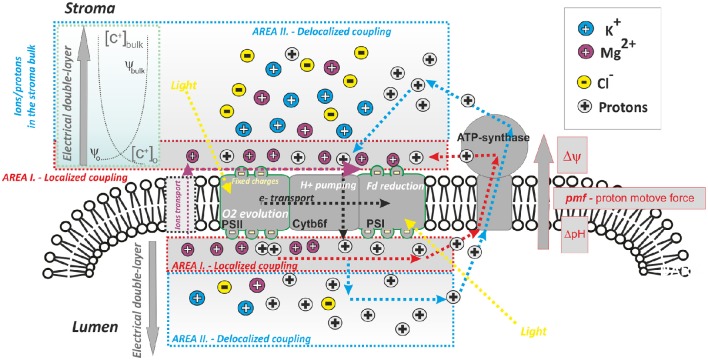
**A simplified scheme of photophosphorylation in photosynthesis: role of proton and ion heterogeneity**. ATP synthase uses proton gradient across the thylakoid membrane; the proton transport across the thylakoid is coupled to light-driven electron transport (dotted black lines). The proton gradient forms electrochemical gradient of protons [*pmf* —proton motive force, which includes Δψ (the membrane potential component) and ΔpH (proton gradient component; see Equation 1)]; *pmf* is the driving force for ATP synthesis. The most accepted view of photophosphorylation is: “delocalized phosphorylation”—equilibrated concentration of protons in the bulk (in the stroma/lumen) is used (original Mitchell theory—dashed blue lines, see Mitchell, 1966). A second less accepted view is that of “localized phosphorylation” (dashed red lines, see Mulkidjanian et al., 2006): protons from the local domains in lumen/stroma that are in the close vicinity of membrane (see Area I) are involved in ATP synthesis (Dilley, 2004). Localized protons interact with ions attached to fixed membrane charges, mostly Mg^2+^ (note: K^+^ and Cl^−^ are more abundant in the thylakoid stroma/lumen bulk, see Barber, 1980b). The distribution of the dominant ions (K^+^, Mg^2+^, Cl^−^) in the local (Area I) and bulk domain (Area II) areas is controlled by properties of the electrical double layer—EDL (see Cevc, 1990); EDL is characterized by the ion profile around the membrane, as well as the electrical field around the membrane, between ψ_o_ and ψ_bulk_. (cf. Figure 2). Photosynthetic proton pumping into the lumen is accompanied by counter-ion transport from lumen into stroma. The process of electron transport and the use of protons by ATPase can be uncoupled by the addition of various ionophores (nigericin—electroneutral antiporter H^+^/K^+^; valinomycin—K^+^ ionophore; A23187—Mg^2+^) that can disrupt the membrane potential and ion/proton gradients. The efficient activity of the particular ionophore in uncoupling requires the presence of appropriate cationic species at the membrane surface (Barber, 1980b); therefore, their uncoupling ability differs between high & low screening modes when more Mg^2+^ and K^+^ are attached to thylakoid membranes (see Figure 5).

Thylakoids and other energy transducing membranes produce ATP employing transmembrane electrochemical gradient of protons; see original papers proving this concept (Junge and Witt, 1968; Junge et al., 1968, 1970; Schliephake et al., 1968) as well as selected reviews (Junge, 2004, 2013; Junge and Nelson, 2015). The proton gradient is formed by proton pumps and is coupled with light-driven electron transport. Peter Mitchell explained the entire process by his chemiosmotic theory (Mitchell, 1961, 1966), for which he received the 1978 Nobel Prize in Chemistry. In this theory, the unidirectional electrochemical gradient of protons across the membranes, the “proton motive force” (*pmf*), is used for ATP synthesis; this *pmf* (Equation 1) consists of two components: electrical (Δψ—the membrane potential) and chemical (ΔpH—proton gradient across membrane), both of which can be used for ATP synthesis (cf. Figure 1):

$$\begin{array}{l} pmf\left(mV\right)\;=\;\Delta \psi +\frac{2.3RT}{F}\Delta pH \end{array}$$

where, R is the universal gas constant, F the Faraday constant, and T the temperature, in Kelvin (equivalent to temperature in Celsius plus 273.15). In mitochondria, Δψ predominates (Mitchell, 1966), whereas in chloroplasts, ΔpH is the major component (Ort and Melandri, 1982). The ΔpH across the membranes is used not only for making ATP from ADP and Pi, but is also known to control the efficiency of light harvesting and photoprotection (Wraight and Crofts, 1970; Briantais et al., 1979) and regulates electron transport and ATP synthesis (Tikhonov, 2013).

**Figure 2:**
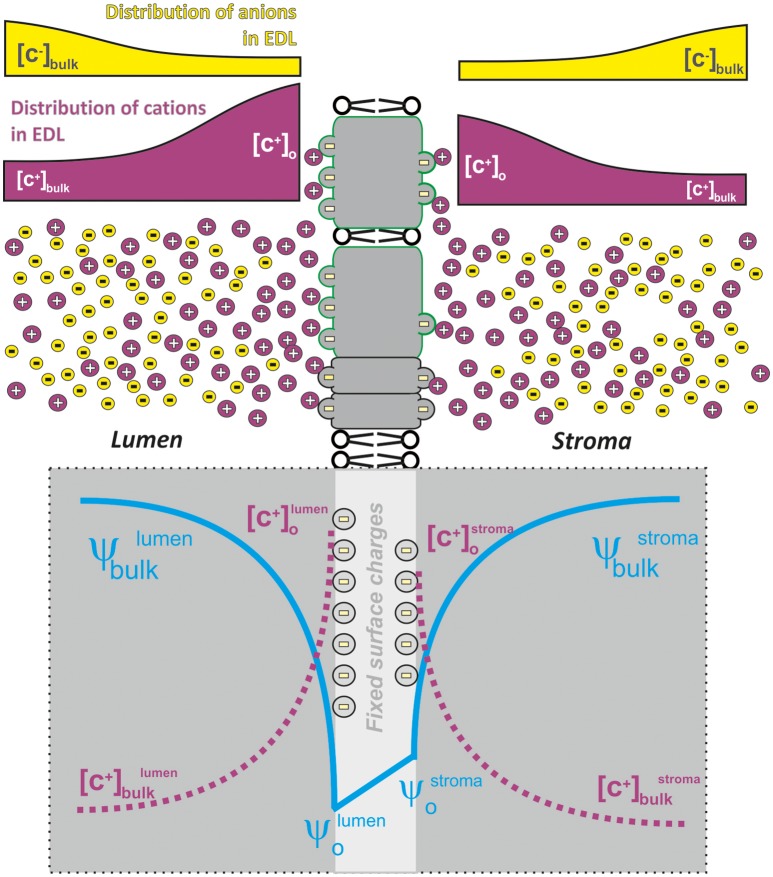
**A schematic representation of charge/electrostatic field distribution around thylakoid membranes that forms electric double layer (EDL)**. Negatively charged amino acid residues form fixed charges on the thylakoid membrane surface that is screened by positive ions (mostly Mg ^2+^) in the lumen as well as in the stroma. The concentration of screening cations decreases with distance from the membrane surface in contrast to that of the anions that are more abundant farther from the negative charges of the membrane. This charge distribution then forms the electric double bilayer (EDL) where the electrical field (see blue line) on the luminal/stromal surface of thylakoid membrane (ψ_o_-lumen, ψ_o_-stroma) is higher than the electrical field measured in the bulk (ψ_bulk_^lumen^; ψ_bulk_^stroma^); there is also a characteristic distribution (see dotted magenta lines) in the concentration of cations at the membrane [C^+^]_o_ and in the bulk [C^+^]_bulk_. EDL then represents asymmetric charge distribution of both the anions (e.g., Cl^−^) and the cations (K^+^; Mg^2+^) caused by fixed charges on the thylakoid membrane; the concentration of the anions/cations progressively increases/decreases with the distance from the charged thylakoid membrane surface (Barber et al., 1977). The difference between ψ_bulk_-ψ_o_ is characterized by the extent of electrostatic screening—the higher the electrostatic screening by the ions the lower is the observed difference.

There is still no clear consensus about the precise division of steady-state thylakoid proton motive force between the ΔpH and the electric field gradient (Δψ). Based on measurements of electrochromic shift, there are irreconcilable findings indicating a partial (Cruz et al., 2001), or a total collapse of Δψ (Johnson and Ruban, 2014) under steady state conditions. To resolve this discrepancy, we need to know the extent of particular counter-ion transport (i.e., K^+^, Cl^−^, Mg^2+^) in the steady state (see discussion Kramer et al., 1999; Cruz et al., 2001) as the transport of both protons and ions affect the partitioning of *pmf* into Δψ and ΔpH (Kramer et al., 2004; Lyu and Lazár, 2017). The *pmf* partitioning is an important factor indirectly regulating the light-harvesting: (1) by lumen acidification that triggers non-photochemical quenching (see Horton, 2014); (2) by changes in ion (mostly Mg^2+^) concentration in the stroma, which affects excitation energy redistribution between the two photosystems, during state transitions (see Barber, 1980b). Importantly, the concentration of ions and the presence of electrical fields across the membrane (Δψ) are tightly interconnected as they both form an “electrical double layer”—EDL (see Figures 1, 2). Here, we have a typical heterogeneous structure defined by charges and by electrical fields formed close to the negatively charged membranes (Cevc, 1990). In chloroplasts, both monovalent and divalent cations electrostatically interact with negatively charged surface of the thylakoid membrane proteins (see e.g., Barber, 1980a,b, 1982, 1986, 1989), and both the free ions and the fixed charges of membrane proteins form the EDL of the thylakoid membrane (Figure 2). Any change in heterogeneous EDL affects reactions in photosynthesis (Barber, 1980b).

The original chemiosmotic theory did not include any heterogeneity in ions or in proton concentrations (Mitchell, 1961, 1966). It had suggested that ATP synthesis is driven by equilibrated proton gradient between two homogenous cellular compartments in the bulk aqueous phases (see Figure 1); this type of photophosphorylation is considered to be delocalized. Even though the theory was experimentally confirmed (see e.g., Junge et al., 1968) some experimental data indicated that proton transfer can sometimes proceed much faster between localized proton domains, situated close to the negatively charged membrane surfaces (Figure 1) by the so—called “localized photophosphorylation” (Dilley, 2004; Mulkidjanian et al., 2006). Importance of localized and/or delocalized photophosphorylation at particular physiological conditions needs to be reconsidered (Dilley, 2004). Therefore, it is obvious that the pmf partitioning must be studied with consideration of the heterogeneous EDL membrane (Figures 1, 2). Moreover, theoretical model has shown an effect of H^+^/ATP stoichiometry, ionic strength, and buffering capacity on pmf partitioning (Lyu and Lazár, 2017). There are also some other, alternative models of photophosphorylation proposing higher importance of ions; for example there is a mechano-chemiosmotic photosphorylation model, where ATP synthase is considered as a “*Ca*^2+^ */H*^+^–*K*^+^ *pump-pore enzyme”* (Kasumov et al., 2015). Last but not the least, there is not only heterogeneity in the distribution of protons (localized/delocalized), but also in the cations (monovalent K^+^ and divalent Mg^2+^) that seems to be distributed asymmetrically—K^+^ being mostly in the bulk of the stroma and Mg^2+^ ions being associated closely with the thylakoid membranes due to their divalent charges (see e.g., Barber, 1980b, 1982; references therein).

In summary, protons and ions are distributed heterogeneously in close vicinity of negatively charged thylakoid membrane surface (see Figures 1, 2). The attraction of ions close to the membrane surface results in screening of the electric field generated by the membrane surface charge. We note that ion screening of membrane charges represents an electrostatic interaction, and it differs from direct neutralization of membrane charges by ions (e.g., H^+^ binding to amino acid residues, see Barber, 1980a). The extent of this screening affects photosynthesis in various ways; effects have been observed on, e.g., variable chlorophyll (Chl) *a* fluorescence (Murata, 1969a), through changes in chlorophyll-proteins, in thylakoid membrane (TM) stacking (Barber, 1980a), and in excitation energy redistribution during light—induced state transitions (see e.g., Barber, 1982; Staehelin and Arntzen, 1983; Telfer et al., 1983). The direct binding of some ions (e.g., Zn^2+^ or Li^3+^) or protons at low pH represents a different effect when membrane stacking is not connected with fluorescence changes (Barber, 1980a). In the following, we describe the role of EDL (i.e., membrane screening), charge neutralization on the membrane (i.e., direct ions interaction with the membrane) on variable Chl *a* fluorescence and on the regulation of light-harvesting in state transitions and during non-photochemical quenching.

## Variable chlorophyll *a* fluorescence of photosystem II

**Figure 3:**
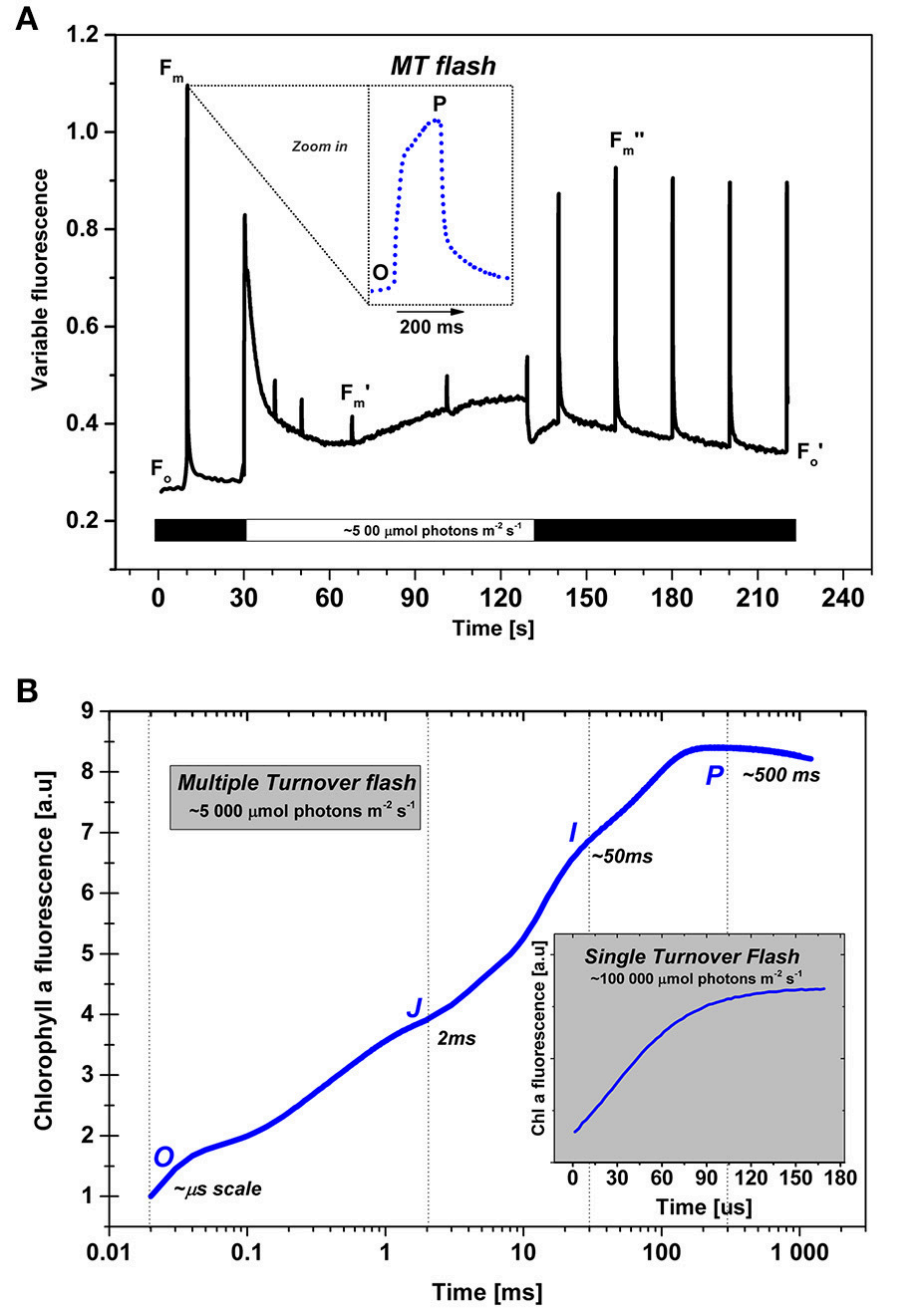
**Chlorophyll a fluorescence measurements in photosynthetic organisms. (A)** (top): A protocol used for the detection of PSII photochemistry upon exposure with ~500 μmol photons m^−2^s^−1^ light (Krause and Weis, 1991). The minimal level of Chl *a* fluorescence of open PSII reaction centers plus that from PSI is F_o_; it is measured in a dark adapted sample at very low (“measuring”) light (~5 μmol photons m^−2^s^−1^). The maximal fluorescence in dark-adapted sample is F_m_, but its changed value during actinic light is referred to as F_m′_, and after the actinic light period light, it is labeled as F_m″_. In the experiment shown, it was measured with high intensity multiple turnover (MT) flashes (~1500 μmol photons m^−2^s^−1^, flash duration 200 ms), given for a short period (~200 ms). (Note: MT flash induces multiple events of charge separation in PSII.) The fluorescence increase during the MT flash has a characteristic polyphasic rise to a plateau or a peak (see inserts in both panels A and B). The F_m_ and F_m′_ (as well as F_m″_) values are used for the calculation of PSII photochemistry as well as for non-photochemical quenching (NPQ) of the excited state of Chl *a*, the latter equals (Fm-Fm′)/Fm. Black bars (near the abscissa) represent periods without actinic irradiation (i.e., darkness), whereas during the open (clear) bar, actinic light is on. The sample used in the experiment shown here was *Rhodomnas salina* cells (from Kaňa et al., 2012b). **(B)** Chl *a* fluorescence transient measured at high intensity (~5000 μmol photons m^−2^s^−1^) MT flash (from Kaňa et al., 2008). The inset shows Chl *a* fluorescence transient in a short, 100 μs long, single turnover (ST) flash, at very high irradiation (~100 000 μmol photons m^−2^s^−1^). Using the MT flash at high light intensity, we observe a polyphasic O-J-I-P fluorescence transient, where the O–J rise is due to primary photochemical reactions, the subsequent J–I–P transient being the thermal phase (cf. Stirbet and Govindjee, 2012). The fluorescence rise in single turnover (ST) flash (see inset in **A**) is the fast O-J-I-P fluorescence change during the single charge separation event induced by the ST flash that closes all the PSII reaction centers in a very short period (in about 30–100 μs) due to the extremely high intensity of light (~100,000 μmol photons m^−2^s^−1^, duration 100 μs). We note that explanation of experimental differences between ST and MT fluorescence parameters, obtained with single and multiple turnover flashes, requires detailed knowledge of the studied model organism (see e.g., Kolber et al., 1998; Koblížek et al., 2001).

The light-harvesting efficiency and the photochemistry in the photosystems (especially Photosystem II, PSII) are often inferred from variable Chl *a* fluorescence measurements (see e.g., chapters in Papageorgiou and Govindjee, 2004). At room temperature, the ratio of variable to minimal chlorophyll *a* fluorescence (F_v_/F_m_ = (F_m_ − F_o_)/F_m_, where F_m_ is the maximal fluorescence for totally closed reaction centers of PSII, F_o_ is the minimal fluorescence at very low excitation intensity, and F_v_ is the variable fluorescence, see Figure 3) has been extensively used as a measure of the efficiency of PSII photochemistry (Krause and Weis, 1991; Govindjee, 2004) since PSI fluorescence is very low and constant (see Giovagnetti et al., 2015; references therein). The kinetic changes in variable chlorophyll *a* fluorescence reflect several processes affected by electron transport, protonation, phosphorylation, NPQ in the antenna, and in PSII reaction centers, as well as in excitation energy redistribution between the two photosystems during state transitions.

Time dependent variable Chl *a* fluorescence intensity changes (fluorescence induction, or fluorescence transient) is measured when dark-adapted cells are exposed to high light; the first measured point is the “O” level, the F_o_, the initial fluorescence (Figure 3). This is followed by an increase to the “P” level (in ms range), due mainly to the reduction of Q_A_ to ${\text{Q}}_{\text{A}}^{-}$. An effect of other processes including electric field, and conformation of proteins is a matter of discussion for the first second after the sample is exposed to light (see e.g., Schansker et al., 2014 for a review). For instance, the non-photochemical thermal (JIP, see Samson et al., 1999) phase can be attributed to the release of quenching of fluorescence associated with light-driven conformational changes in PSII (Schansker et al., 2011), or of “*photoelectrochemical quenching”* controlled by trans-thylakoid proton pump, powered by light-driven Q cycle (see Vredenberg et al., 2009, 2012). The fast Chl *a* fluorescence rise, during the first second of illumination (Figure 3B) is labeled as a O-J-I-P transient (O for the minimum fluorescence, J, and I for inflections, and P for the peak (Strasser et al., 1995; Lazár, 1999, 2006; Stirbet and Govindjee, 2012; Schansker et al., 2014). However, the slower changes in fluorescence (in tens of seconds, to minutes, see Figure 3A) reflect changes in other physiological processes, including state transitions, non-photochemical quenching, and even photoinhibition.

Chlorophyll *a* fluorescence transients, during both fast (~in seconds) and slow (in minutes) time range, have different characteristics in plants, and in cyanobacteria (Ruban and Johnson, 2009; Papageorgiou and Govindjee, 2011; Kaňa et al., 2012a; Kirilovsky et al., 2014). These transients are affected by changes in several factors including: **(a)** the efficiency of PSII photochemistry (reflected by the OJIP phase); (**b**) state transitions (Ruban and Johnson, 2009); **(c)** the coupling and uncoupling of antenna from PS I and or PS II (Kaňa et al., 2009; Kirilovsky et al., 2014); **(d)** photoinhibition of PSII in high light (Prášil et al., 1992); **(e)** lumen acidification during NPQ (e.g., Ruban et al., 2012; Zaks et al., 2013; also see Demmig-Adams et al., 2014); **(f)** the efficiency of carbon cycle reactions; and **(g)** divalent and monovalent ion concentrations that affect EDL, as well as the electric properties of thylakoid membranes (Barber and Mills, 1976; Barber, 1980b, 1982).

The effect of ionic composition of the suspension medium on variable Chl *a* fluorescence of chloroplasts and thylakoid membranes has been intensively studied during the 1970s—1980s (Vandermeulen and Govindjee, 1974; Barber and Mills, 1976). Ion dependent NPQ of Chl *a* fluorescence at room temperature has been examined in green algae (Mohanty et al., 1974), in guard cells of *Vicia faba* (Ogawa et al., 1982), as well as in cyanobacteria (Papageorgiou and Stamatakis, 2004). The effect of Mg^2+^ has also been examined by low temperature (77 K) fluorescence spectra in plants. Further, variable Chl a fluorescence during the J-(I)-P phase (Figure 3B) of fluorescence induction may also be affected, in general, by electric field on the thylakoid membrane, as well as by PSI-dependent photoelectric stimulation and transient release of “*photo-electrochemical quenching”* affected by trans-thylakoid proton pump, which, in turn, also involves the Q cycle (Vredenberg and Bulychev, 2002; Vredenberg et al., 2009, 2012). Even though the Vredenberg model has not been generally accepted, mainly, due to several controversial assumptions (see e.g., Stirbet and Govindjee, 2012), the effect of electrical field and ions on total variable Chl *a* fluorescence must be taken into account in light of other experimental data, i.e., the effect of valinomycin, a K^+^ ionophore, on the thermal phase of Chl *a* fluorescence induction (Pospíšil and Dau, 2002; Antal et al., 2011), and the effect of ions on variable Chl *a* fluorescence *in vivo* (Mohanty et al., 1973, 1974; Ogawa et al., 1982; Papageorgiou and Stamatakis, 2004; Krupenina and Bulychev, 2007). The effect of cations and or electrical field on fluorescence could also be related to the EDL in thylakoids, formed by ions and fixed membrane charges (see Figure 2; Cevc, 1990). Different screening (low/high—i.e., due to electrostatic interactions) of the membrane charges by ions interferes with the light harvesting process in many aspects, and it is clearly seen through changes of variable fluorescence during organization (or reorganization) of thylakoid membranes (Barber, 1980a,b, 1982, 1986, 1989).

## The electrical double layer (EDL) and the thylakoid membrane

The electrical double layer (EDL) represents a typical structure formed when (biological) membrane surfaces, with fixed negative charges on them, are in contact with an aqueous medium containing cations (Cevc, 1990); here, then, we have asymmetric charge distribution of ions that progressively increases toward electrically charged surfaces, i.e., thylakoids (Figure 2). The definition of EDL is based on changes of electrical field (ψ_o_ a parameter measurable by electrochromic shift (Cruz et al., 2001) and ion concentration ([C^+^] with increasing distance from the membrane; Figure 2). In fact, ions located close to the membrane surface result in *screening* of the electric field of the membrane surface charge (i.e., damping of electrostatic field of fixed charges caused by the presence of interacting ions). The term screening in this case describes the ability of ions in the aqueous phase to increase the negative surface potential closer to zero (see ψ_o_ in Figure 2; for details and equations, see e.g., Barber, 1980b) and it is different than direct interaction of ions with the membranes (i.e., charge neutralization, see Barber, 1980a).

The thylakoid membrane is negatively charged at physiological pH (see reviews Barber, 1980b, 1982; references therein). The average area per single electronic charge on the thylakoid membrane has been estimated to be about 10 nm^2^ (Barber, 1980b). Based on isoelectric measurements on isolated thylakoids (Åkerlund et al., 1979), the total fixed negative charges on thylakoid membranes have been estimated to be higher on their luminal, than on their stromal, surface. There are also some experiments indicating an increase in the fixed negative charge during irradiation (see discussion in Barber, 1982) and reference therein). These charges can be attributed to the carboxyl groups of glutamic and aspartic acids in the pigment protein complexes that are negatively charged at the physiological pH (cf. Behrens et al., 2013). An involvement of negatively charged lipids (sulfoquinovosyldiacylglycerol and phosphatidylglycerol) in the total charge, on the thylakoid membrane, is still unclear since these lipids represent only about 20% of all the lipids and they are mostly the boundary lipids for the membrane proteins or act as their cofactors (see e.g., Mizusawa and Wada, 2012). However, the effect of anionic lipids and negatively charged domains of chlorophyll-containing proteins on antenna aggregation/dis-aggregation is indeed a reality (Schaller et al., 2011, 2014). Therefore, the involvement of negatively charged lipids in the total thylakoid membrane charges and on the organization of membrane proteins needs to be further explored.

**Figure 4:**
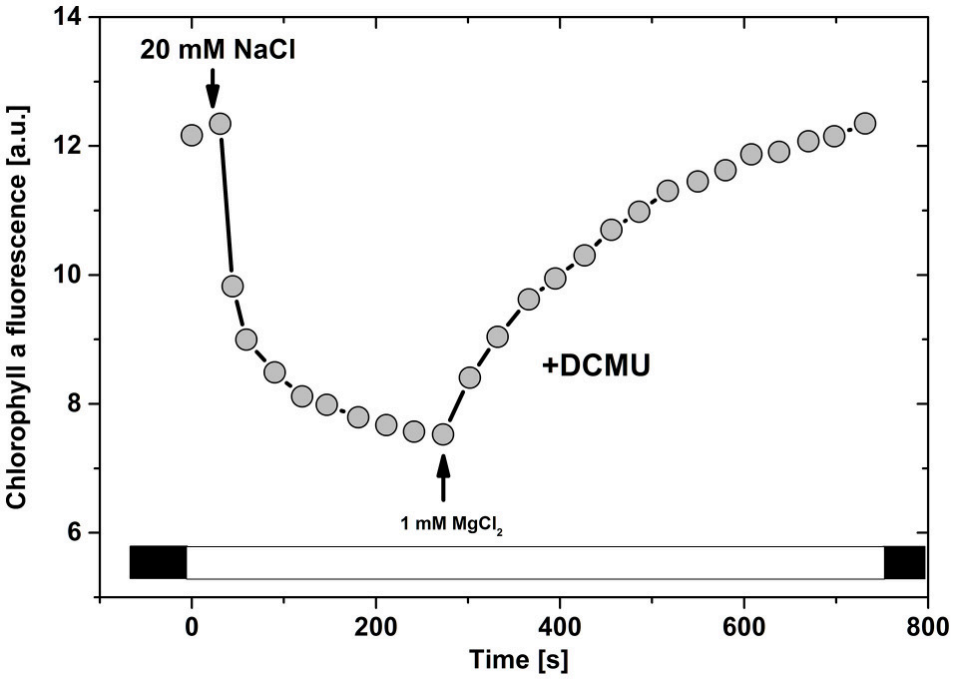
**Ion induced changes in variable chlorophyll *a* fluorescence in isolated thylakoids (data adapted from Vandermeulen and Govindjee, 1974)**. Time course of cation induced changes in chlorophyll *a* fluorescence in oat chloroplast suspension containing 5 μg chlorophyll ml^−1^, and 5 μM DCMU in low salt buffer. Subsequently, 20 mM NaCl and 1 mM MgCl_2_ were added (see arrows).

The EDL in photosynthesis was initially examined for changes in variable Chl *a* fluorescence intensity at different ion concentrations (Barber and Mills, 1976; Figure 4). This effect is independent of PSII activity since it was measured with PSII inhibited by DCMU (Gross and Hess, 1973). In the simplest model, monovalent (K+, Na+) and divalent (Mg^2+^ Ca^2+^) ions have antagonistic effects on chlorophyll a fluorescence (Gross and Hess, 1973; Wong and Govindjee, 1979). In the low salt medium, Chl *a* fluorescence is inhibited by monovalent ions (~5 mM of K^+^); however, this inhibition is reversed by the addition of divalent ions (e.g., ~5 mM of Mg^2^ see e.g., Figure 4B). The decrease in Chl fluorescence intensity by monovalent cations has been rationalized by the presence of divalent cations on the membrane surface, before the addition of monovalent ions (Nakatani et al., 1978); the addition of monovalent ions causes their exchange with divalent ions, and Chl *a* fluorescence decreases because monovalent ions have lower capacity of electrostatic screening (Barber, 1989). This effect has been found to be even more complex since ~5 mM K^+^ can reduce Chl *a* fluorescence in the presence of low Mg^2+^ (about 0.1 mM, see Mills et al., 1976), but the high concentration (e.g., ~50 mM) of monovalent K^+^ has been shown to enhance variable Chl fluorescence just as low divalent Mg^2+^ does (Barber et al., 1977; Mills and Barber, 1978). Therefore, the conclusion is that the maximal variable fluorescence can be observed only when positive charge density on the thylakoid membrane surface is sufficiently high and above a critical value (Barber and Mills, 1976).

This dependence of fluorescence on ion concentrations is based only on the valence of interacting ions: a similar effect is observed for K^+^ and Na^+^, which is different for divalent Mg^2+^ and Ca^2+^ (Nakatani et al., 1978); further, there is almost no specificity in ion-protein and in ion-lipid interactions for particular ions (Mills and Barber, 1978). This phenomenon can be thus associated with a delicate interplay of mixed “electrolytes” (e.g., in lumen, or stroma) containing a mix of ions (e.g., K^+^, Mg^2+^, Cl^−^, and Ca^2+^) with fixed negative charges of proteins (or lipids) on the thylakoid membranes. These ions can then electrostatically interact with negative charges on thylakoid membranes, thus influencing photosynthesis (Barber et al., 1977). The above-described phenomenon has been theoretically explained by James Barber, and his coworkers, using a modified Gouy-Chapman theory, including non-linear Poisson-Boltzmann ion distribution (Barber et al., 1977). This theoretical approach provided an electrochemical model for EDL on thylakoid membranes at different ion concentrations, and simulated further research in this area (Barber et al., 1977; Rubin and Barber, 1980; Barber, 1989). We can now write several conclusions and caveats: **(a)** Much lower concentration of divalent cations is required in the bulk solution compared with that of the monovalent cations to provide the same extent of electrostatic screening, as reflected in the surface potential (ψ_o_); **(b)** The way in which positive charges distribute within the diffuse layer is different for divalent and monovalent cations; **(c)** The concentration of anions in the diffuse layer is very low. Thus, the observed different effects of particular ions (K^+^, Mg^2+^, Cl^−^, and Ca^2+^) on variable Chl *a* fluorescence can be explained by a pure electrochemical effect, due to their different electrostatic screening of charges of membrane proteins (Rubin and Barber, 1980). We note that the effect, discussed here, does not include direct cation binding on membranes (Barber, 1980a). The direct and unspecific cation binding to negatively charged residues could not be totally ruled out (Mills and Barber, 1978; Barber, 1980a). However, in the case of the direct ion binding to membranes (e.g., for La^3+^, Zn^2+^ or for protons at pH 4.3) no fluorescence changes are observed (Figure 4), but only membrane stacking (Berg et al., 1974; Barber and Searle, 1978; Mills and Barber, 1978; Gerola et al., 1979; Barber et al., 1980; Chow and Barber, 1980). Therefore, Mills and Barber (1978) and Barber (1980a) concluded that electrostatic forces (i.e., effect of EDL) are responsible for reversible fluorescence changes connected with membrane re-organization.

**Figure 5:**
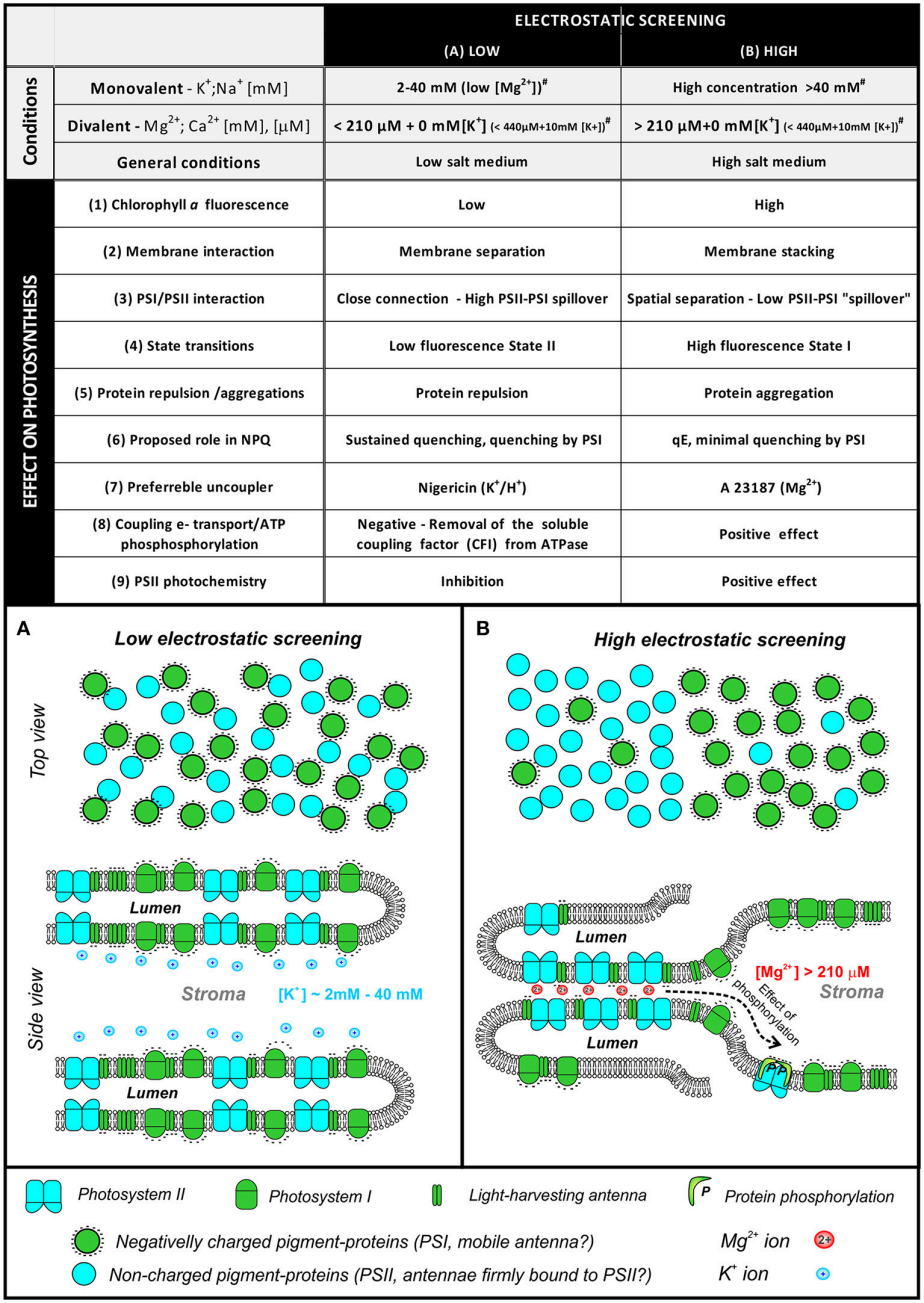
**A simplified scheme of changes in protein organization in different states of electrostatic screening (adapted from Barber, 1980b)**. The table summarizes basic conditions for low or high screening—high screening is induced by high divalent cation content [e.g., >220 μM Mg^2+^ with no (K^+^), >440 μM Mg^2+^ with 10 mM (K^+^), see (Mills and Barber, 1978) for details]. The switch between low/high screening mode is a complex interplay between monovalent/divalent cation concentrations as it is described by EDL model of thylakoid membrane (Barber et al., 1977). The low/high screening state is then reflected in various physiological processes including intensity of Chl *a* fluorescence (low intensity /high intensity), thylakoid membrane stacking or separation, lower/high excitation spillover between photosystems, State II/State I, protein repulsion/aggregation, proposed role in non-photochemical quenching, preferable uncouplers (Mg^2+^ ionophore A23187 vs. K^+^/H^+^ antiporter nigericin), effect on electron-proton coupling in photophosphorylation, effect on PSII photochemistry. The screening is caused by ion attachment to membranes that results in screening of the electric-field of the membrane charge (i.e., damping of electrostatic field of fixed charges caused by the presence of interacting ions). **(A)**. Simplified scheme of membrane protein organization in the state of low electrostatic screening. Green cycles represent negatively charged proteins (PSI complex, light harvesting antennas), cyan particles represent less charged PSII. Monovalent cation K^+^ is shown as a dominant ion attached to the negatively charged membrane surface. **(B)** A simplified scheme of membrane protein organization in the state of high electrostatic screening, divalent cation, Mg^2+^, is proposed to be the main ion attached to the negatively charged membrane surface. Role of protein phosphorylation on protein redistribution is indicated (^#^ values were taken from Mills and Barber, 1978).

Different concentrations of monovalent and divalent ions (and their different mixtures) affect EDL properties and produce not only changes in Chl *a* fluorescence (Figure 4), but they also affect many other photosynthetic processes (see Figure 5 and reviews by: Barber, 1982, 1986, 1989 and Stys, 1995). In the simplest model, we define two extreme states that are characterized by high or poor electrostatic screening (Figure 5). These two extreme states are defined not only by their particular ion concentration, but by their combined effect on ψ_o_ (see e.g., Rubin and Barber, 1980; Barber, 1989). In view of this, the thylakoid membrane seems to be in the same state with “high electrostatic screening” under the following conditions (Figure 5): (**i**) Divalent cations in the diffuse layer, with a low concentration of monovalent cation in the bulk medium; (**ii**) Very high concentration of monovalent cations (e.g., >50 mM of K^+^) in the bulk medium (Barber, 1986). Further high electrostatic screening is found (see Figure 5) under the following conditions: (**1**) In stacked thylakoid membranes at high Mg^2+^ (Izawa and Good, 1966) since membrane surface charges regulate membrane-membrane interaction; (**2**) When there is an increase in Chl *a* fluorescence yield (Gross and Hess, 1973; Vandermeulen and Govindjee, 1974); (**3**) When there is a decrease in the “*spillover*” of excitation energy from Photosystem II (PS II) to Photosystem I (PS I) (Murata, 1969a)—separation of PSI and PSII into domains; (**4**) When there is a transition to high-fluorescence State I (more antenna coupled to PSII); (**5**) When there is high protein aggregation of antennas and photosystems (we note that protein aggregation, based on current model of NPQ, leads to a quenched state of antennas with low fluorescence yield—this discrepancy needs to be solved by future experiments); (**6**) In isolated thylakoids, A23187, a Mg^2+^/Ca^2+^ ionophore, is a better uncoupler under high, than low, electrostatic screening mode; the uncoupler activity of all the ionophores (including nigericin—H^+^/K^+^ antiporter, valinomycin—K^+^ carrier, gramicidin—cation ionophore, and A23187—Mg^2+^ ionophore) is dependent on adequate supply of appropriate cations at the membrane surface (Barber, 1980b); (**7**) At high-electrostatic screening mode, there is minimal quenching of fluorescence by “spillover” of excitation energy from PSII to PSI; (**8**) There is positive coupling of electron transport with phosphorylation (Walz et al., 1971); (**9**) Higher PSII efficiency at high screening mode is due to a positive effect on water oxidation and reduction of plastoquinone (Dau and Sauer, 1991; Karge et al., 2014; Khan et al., 2015). In conclusion, the switch between the low and the high electrostatic screening is caused by changes in ions and their concentration. This process affects light-harvesting efficiency in photosynthesis, especially during state transitions, or during excitation energy spillover, as monitored by Chl *a* fluorescence. In addition, there is also an effect on the NPQ of the excited state of Chl *a* (Mills et al., 1976). In view of the above observations, we describe below effects of ions on some of these processes.

## Redistribution of excitation energy between the photosystems, membrane stacking and the role of ions in state transitions

The redistribution of absorbed light between the two photosystems is controlled by state transitions, a process described independently by Bonaventura and Myers (1969) in green algae and by Murata (1969b) in red algae, and discussed soon thereafter by Duysens (1972). This phenomenon was also described earlier, without being named as such, by Papageorgiou and Govindjee (1968a,b), as recognized by Krause and Jahns (2004). This process defined as state transitions has been studied in details in cyanobacteria, green algae, and in higher plants (Ruban and Johnson, 2009; Papageorgiou and Govindjee, 2011; Kirilovsky et al., 2014; Nagy et al., 2014; Kodru et al., 2015). In contrast, fluorescence changes usually attributed to state transitions (see e.g., Allen and Mullineaux, 2004) seem to be absent in algae from the red evolutionary clade (excluding the cryptophytes Cheregi et al., 2015) and the red algae (Kaňa et al., 2014). The basic function of state transitions is redistribution of the absorbed light between the two photosystems (PSI and PSII) which optimizes the overall electron transport since the two systems operate in series (Govindjee and Björn, 2012).

*In vivo*, the two light-induced states have been defined as: (**1**) State I—characterized by an increased effective light absorption cross-section of PSII (i.e., a bigger PSII antenna); it is called State 1 because it is induced by light preferentially absorbed by PSI; (**2**) State II—characterized by an increased effective cross-section of PSI (bigger PSI antenna); it is called State II because it is induced by light preferentially absorbed by PSII antenna (Duysens, 1972; Goldschmidt-Clermont and Bassi, 2015). At room temperature, Chl *a* fluorescence is high in State I, but in State II, it is low since the quantum yield of PS II fluorescence is much higher than that of PSI due, in part, to the higher rate of photochemistry in PSI than in PSII and persistent quenching of PSI fluorescence by P700. The single unmistakable way to differentiate between State I and State II in green plants, green algae and in cyanobacteria is to compare fluorescence spectra at 77 K. In State I in plants, far-red PS I emission at 735 nm (F735) is lower compared to that from PSII at 685 nm (F685), whereas in State II, it is the opposite (Allen and Mullineaux, 2004). We note that in organisms that lack a well-separated far-red emission of PSI [e.g., in red-clade alga *Chromera velia* (Belgio et al., 2017) and in the dinoflagellate Gonyaulax polyedra (Govindjee et al., 1979)], this method cannot be used.

In addition to state transitions described above for intact cells, ions regulate excitation transfer between photosystems (see e.g., Murata, 1969a; Wong et al., 1979). In fact, fluorescence changes typical for State I and State II can also be induced in isolated chloroplasts/thylakoids that are exposed to different ionic composition (see Figure 4): (**1**) When exposed to high [Mg^2+^], thylakoids in higher fluorescence State I not only have higher PSII antenna size, but their membranes are in high electrostatic screening mode (Murata, 1969a), and are highly stacked; (**2**) Low fluorescent State II occurs if the concentration Mg^2+^ cation is lowered to very low values (below 100 μM, without any other cation like K^+^, see Mills and Barber, 1978) or in the presence of monovalent cations (e.g., ~5 mM K^+^) in ion—free medium; in this case, a decrease in PSII fluorescence is considered to be due to State I to State II transition (see Figure 4). We note that milimolar [K^+^] and [Mg^2+^], given alone, have opposite effects on fluorescence (K^+^ ions decrease, and Mg^2+^ ions increases fluorescence). However, if they are given together, the effect is more complex (for details, see Mills and Barber, 1978). The fluorescence decrease can be also induced by anions; higher effects are observed with increasing valence (Jajoo et al., 1998, 2001). Therefore, it is clear that anions (Jajoo et al., 1998; Jajoo and Bharti, 1999) and monovalent cations can reverse the effect of Mg^2+^ that switches thylakoid membranes into a high electrostatic screening mode (see Figure 5). These negative charges act on the outer side of the thylakoid membrane due to the electrostatic interaction with the negative charges of proteins, and this affects the extent of stacking of thylakoid membranes (Barber, 1982).

As a matter of fact, state transitions are triggered by redox shifts of the PQ-pool [the PQ-pool is more reduced in State II (Mullineaux and Allen, 1986)]; in higher plants and in green algae, it is accompanied by phosphorylation of a mobile light-harvesting complex (in State II) (Horton and Black, 1980; Allen et al., 1981; Tikkanen and Aro, 2012). This occurs on the outer surface of thylakoid membranes (for further information, see Vener et al., 2001; Vener, 2007), and contributes to the total negative charge of the outer thylakoid membrane surface. There is a specific STN7 kinase that phosphorylates light harvesting antenna of PSII—LHCII, which is then redistributed to PSI (Bellafiore et al., 2005) during state I to state II transition. On the other hand, another kinase, Stn8, phosphorylates PSII subunits and is involved in PSII repair during photoinhibition (Tikkanen et al., 2008). The current model of state transitions in higher plants involves phosphorylation which leads to addition of negative charges on the mobile LHCIIb phosphate groups provide those charges; this is in agreement with the electrostatic screening model of state transitions that includes a role of negative charges on the pigment proteins (Barber, 1980b, 1982; Staehelin and Arntzen, 1983; Stys, 1995). Indeed, the higher mobility of phosphorylated (mobile) LHCII has been confirmed (see Consoli et al., 2005); see reviews by Mullineaux (2008), Kaňa (2013), Kirchhoff (2014). The connection between electrostatic screening, protein phosphorylation, protein redistribution and membrane organization (stacking/de-stacking) is still not clear; in all likelihood, phosphorylation of PSII in plants exposed to high light enhances the stacking dynamics of the photosynthetic membranes (Barber, 1982; Fristedt et al., 2010).

The molecular mechanism of excitation energy redistribution during state transitions is either due to antenna (LHC II) redistribution between the two photosystems, or a change in PSI/PSII interaction. The latter causes differences in “excitation energy spill over” from one system to another (from the “slower” PSII to the “faster” PSI, see e.g., Mirkovic et al. (2016) where PSI acts as an efficient fluorescence quencher (Barber, 1982; Slavov et al., 2013). In some cases, the antenna uncoupled from one photosystem (e.g., from PSII) is not necessarily coupled to the other photosystem (e.g., PSI) and remain(s) uncoupled in the membrane (Kaňa et al., 2009; Kirilovsky et al., 2014; Unlu et al., 2014; Cheregi et al., 2015). The role of these uncoupled antennas needs to be taken into account in any future model of state transitions (Goldschmidt-Clermont and Bassi, 2015). Based on the correlation between state transitions, membrane stacking and ion composition, and interconnection between state transition mechanism and changes in electrostatic screening (Barber, 1980a), we may very well consider combining the phenomenon of electrostatic screening with the current model of state transitions in higher plants that requires getting a negative charge through phosphorylation (Tikkanen and Aro, 2012). We note that phosphorylation can have a dramatic effect on the membrane surface charge as there are multiple phosphorylation sites on PS II, and each phosphorylation provides two negative charges at a stromal pH of 8.0. Further experiments and analysis are needed to reach this goal.

The molecular mechanism of state transitions representing antenna redistribution and/or change in excitation energy spillover between PSII/PSI is caused by a variation in the balance between electrostatic repulsion (coulombic repulsive forces between negative charges of proteins affected by electrostatic screening) and protein attraction caused by van der Waals forces that are independent of charges on the proteins (see e.g. Barber, 1980b; Stys, 1995). The higher/lower electrostatic screening of negatively charged proteins by ions affects the balance between protein repulsion (low electrostatic screening, Figure 4) and attraction (high electrostatic screening). This concept is based on **D**erjaguin-**L**andau-**V**ervey-**O**verbeek (DLVO) theory for the aggregation of colloids—more divalent cations lead to membrane stacking due to reduction in electrical repulsion (Barber, 1982; Stys, 1995). This process occurs *in vitro* when changes in [Mg^2+^] affect PSII LHCII interactions (see e.g., Kiss et al., 2008). Moreover, membrane stacking can be induced also by lowering of stromal pH to about 5.4 inducing electrostatic neutralization (i.e., by increasing [H^+^]), which is closer to the isoelectric point of the thylakoid membrane (Gerola et al., 1979; Jennings et al., 1979). The stimulating effect of low pH on stacking has also been shown in the light-induced spontaneous tendency of thylakoid membranes to stack (Janik et al., 2013). Proteins can lose charges when the pH of the medium is close to the pKs of their negatively charged amino acids (Nakatani and Barber, 1980; Behrens et al., 2013); close to their pKs, proteins become electroneutral. However, in this case membrane stacking is usually caused by charge neutralization, not by charge shielding through EDL. Moreover, this effect is not connected with protein redistribution and with fluorescence changes (Barber, 1980a).

However, when thylakoid membranes stack, Chl *a* fluorescence increases, and State I induced by high Mg^2+^ (high electrostatic screening state, see Figure 5) usually appears simultaneously (Barber, 1980a; Barber et al., 1980). In this model, which is based on EDL, ion-dependent effects on antenna proteins are single regulatory events [e.g., re-arrangement of different super complexes at different [Mg^2+^] inducing stacking of thylakoid membranes leading to changes in Chl *a* fluorescence (Rumak et al., 2010)]. However, there are some special cases when they occur independently of each other, usually when direct charge neutralization (direct ion/proton binding) occurs (Mills and Barber, 1978; Barber, 1980a). An example is Mg^2+^ induced antenna coupling with PSII, without membrane stacking (Kiss et al., 2008); whether this is because of electronic screening, or neutralization, needs to be ascertained (Mills and Barber, 1978; Barber, 1980a; Scoufflaire et al., 1982). However, we speculate that these ion-induced effects (membrane stacking/fluorescence changes) are controlled by two different mechanisms that usually, but not always, co-occur.

In fact, the two independent processes induced by Mg^2+^ addition have also been shown to exist through fluorescence measurements (Jennings et al., 1982). In one case, an ion effect has been shown to work only on the stromal side (e.g., by the application of impermeable poly L-lysine): there was restacking without fluorescence increase (Berg et al., 1974). Moreover, it has been clearly shown that ion-induced thylakoid stacking/unstacking in the grana region can appear without any change in variable Chl *a* fluorescence, and without any change in the connectivity between different units of antenna (Kirchhoff et al., 2004), again depending on whether electrostatic screening or neutralization is present (Mills and Barber, 1978; Barber, 1980a; Scoufflaire et al., 1982). On the contrary, these phenomena are found to be connected in thylakoids (granal and stromal membranes) indicating the importance of stroma for thylakoid membrane reorganization (Kirchhoff et al., 2004). This indicates a role of a stromal factor, or a requirement of *less-protein-crowded* stromal thylakoids for grana reorganization. Based on the hypothesis proposed by Barber (1980a), the membrane stacking connected with fluorescence changes represents a switch between high/low electrostatic screening (i.e., it is an effect of electrostatic interaction—EDL); the disconnection between fluorescence and membrane stacking is caused by the presence of membrane charge neutralization, which means direct interaction of ions (or protons) with membrane charges. There are also some other experimental data indicating that proton/ion regulation of light-harvesting requires at least two independent regulatory events (Wollman and Diner, 1980; Jennings et al., 1982; Kirchhoff et al., 2003, 2004; Stoitchkova et al., 2006; Kiss et al., 2008); plausible candidates for these effects could be screening or binding of ions (Barber, 1980a). However, to explore conditions, when these processes (i.e., membrane stacking and state transitions) are independent, additional experiments affecting the ratio of ion screening/ion binding are needed to confirm the proposed concepts.

The mechanism of state transition(s) requires a certain reorganization of thylakoid membrane proteins. The positive effect of protein phosphorylation on structural flexibility of the thylakoid membrane architecture has been confirmed (Varkonyi et al., 2009). Thylakoid membrane proteins are also differently organized (random or into domains) for different electrostatic screening (low or high; see Figure 4; Barber, 1982). The movement of these differently charged proteins is induced by lateral charge displacement on the outer side of the thylakoid membranes (see e.g., Barber, 1986); this can be induced either by protein phosphorylation higher mobility of phosphorylated (mobile) LHCII (Consoli et al., 2005) or by some unspecific effects of ions causing columbic repulsion between PSII and PSI. We note that only with screening (i.e., electrostatic effect of ions without interaction), we have lateral diffusion of PSI (in stroma lamellae) and of PSII (in grana), as well as changes in spillover from PSII to PSI. With neutralization of surface charges by ion binding (e.g., protonation at low pH, Barber et al., 1980), the membranes simply collapse on each other to give a grana-like appearance with no lateral separation of PSI and PSII. Therefore, increase in the electrostatic screening leads to the formation of heterogeneous domains of low-charge/high-charge, resulting in fluorescence changes (Barber et al., 1980).

## Regulatory role of protons and ions in triggering non-photochemical quenching of the chlorophyll excited state

Non-photochemical quenching (NPQ) of the excited state of chlorophyll *a* is a process that protects PSII against excess light (Ruban et al., 2012; Zaks et al., 2013; Croce and Van Amerongen, 2014); for further details see Demmig-Adams et al. (2014) by stimulating the dissipation of excessive irradiation into heat (Kaňa and Vass, 2008). NPQ significantly reduces the quantum yield of variable fluorescence (by even 60%, see Ostroumov et al., 2014) and affects the efficiency of energy transfer from the antenna to the reaction centers (see review on the energy-transfer dynamics in photosynthesis Mirkovic et al., 2016). In higher plants, NPQ occurs mainly in the light-harvesting antenna (Gilmore et al., 1995; Horton et al., 1996; Belgio et al., 2012). However, in cold tolerant plants or in certain extremophiles (algae or cyanobacteria), closed PSIIs can also act as quenchers (see e.g., Ivanov et al., 2008; Krupnik et al., 2013). Kinetically, NPQ is divided into at least three major components: (**1**) the “energy dependent” quenching (qE), which is triggered by a faster (<1 min) light-driven proton translocation across the thylakoid membrane (Barber, 1976; Krause et al., 1983); (**2**) a slower, less dominant, quenching component that has been attributed to state transitions (qT—Allen et al., 1981); and (**3**) slowest components, such as zeaxanthin-dependent quenching, qZ (Nilkens et al., 2010; Ocampo-Alvarez et al., 2013), and a photoinhibitory quenching, qI (Krause, 1988), more generally, simply a “sustained quenching” (Ruban and Horton, 1995).

The triggering role of protons in qE activation was proposed for the first time by Wraight and Crofts (1970) for cyclic electron flow in samples where PSII activity was inhibited by DCMU, and diaminodurene (DAD), a mediator of PSI-dependent electron transport stimulating ΔpH, was added (see discussion in Papageorgiou and Govindjee, 2011). The triggering of qE by luminal protons *in vivo* was established directly by pH titration in isolated chloroplasts (Briantais et al., 1979). Finally, a direct protonation of pigment-proteins has been observed *in vitro* (Ruban et al., 1994; Walters et al., 1996; Kaňa et al., 2012b; Xiao et al., 2012; Belgio et al., 2013). The qE sensitivity to luminal protons is controlled by various allosteric regulators (see the details of the concept in Ruban et al., 2012) including xanthophylls (e.g., zeaxanthin, and violaxanthin Niyogi et al., 1998; Kaňa et al., 2016) and the PsbS protein (see e.g., Li et al., 2000; Johnson and Ruban, 2010).

The co-regulation of low pH-induced qE with ions, and with electrostatic screening of thylakoids, is not clear. Noctor et al. (1993) have shown that a relatively high concentration of Mg^2+^ (about 10 mM) is necessary to obtain maximal qE in isolated thylakoids. This Mg^2+^-dependent mechanism for NPQ (probably related to quenching by PSI, see below) can be also induced by changes in Mg^2+^ concentration in the dark (Briantais et al., 1979; Krause et al., 1983). This could indicate that the maximal extent of flexible qE requires high electrostatic screening of thylakoid membrane charges, which means a higher content of Mg^2+^ on the thylakoid surface (see Figure 5). However, some specific effects of ions on antenna aggregation, and, thus, fluorescence quenching, cannot be totally excluded. Mills et al. (1976) have suggested existence of a cation-sensitive site influencing fluorescence on the stromal surface of thylakoids, based on a similar effect of impermeable choline and K^+^ on fluorescence decrease. A possible ionic effect of K^+^ has also been shown for the “slow” component of NPQ based on experiments with added valinomycin, a specific K^+^ ionophore (Sokolove and Marsho, 1979). However, this data, seemingly, contradicts earlier data showing that qE is sensitive only to K^+^/H^+^ antiporter—nigericin, but not inhibited by valinomycin (Wraight and Crofts, 1970). Therefore, further experiments that would more carefully consider screening mode (high/low—see Figure 5) are needed to resolve the above-mentioned discrepancy.

Ion concentration can also affect Chl *a* fluorescence quenching *in vivo* (Mohanty et al., 1974; Ogawa et al., 1982); however, the mechanism of its effect is not yet clear. Further, we could propose a different mechanism for qE-triggering at low electrostatic screening of thylakoid membrane charges (see Figure 5) when spillover of excitation energy from PSII to PSI may be increasing—in this case, PSI may be acting as a fluorescence quencher. A similar mechanism for NPQ has been proposed for desiccating mosses and lichens (Yamakawa et al., 2012; Slavov et al., 2013). This could be a typical photoprotective NPQ mechanism during desiccation (Bilger, 2014) or in certain algae it could be due to a high spillover of energy from PSII to PSI (see e.g., data obtained with algae *C. velia* Quigg et al., 2012; Kotabová et al., 2014). Further, data of Ruban and Horton (1995) indicate that pH-independent fraction of sustained quenching can be inhibited by the addition of nigericin, a K^+^/H^+^ uncoupler. One could speculate that both, i.e., quenching by PSI (due to spill over of excitation energy from PSII to PSI) and pH independent fraction of sustained quenching are present at low electrostatic screening mode (see Figure 5). However, to confirm these hypotheses, further experiments are needed.

We suggest that the co-regulation of NPQ by both pH and ions (e.g., by Mg^2+^) is indicated by Mg^2+^ counter ion transport upon exposure of photosynthetic samples to light (Figure 1) since the accumulation of protons in the lumen is balanced by the efflux of Mg^2+^ ions to the surface of the thylakoid membrane (Hind et al., 1974; Chow et al., 1976; Ishijima et al., 2003). Moreover, there is data showing the presence of Mg^2+^ transporter in the chloroplast (Drummond et al., 2006) and light induced changes in Mg^2+^ concentration (Ishijima et al., 2003). The stimulating role of Mg^2+^ on protein aggregation *in vitro* is well known: higher Mg^2+^ content accelerates reversible quenching of Chl *a* fluorescence in isolated antenna by forming aggregates of LHCIIs *in vitro* (Ruban et al., 1994). This supports the idea that antenna aggregation is a plausible mechanism for qE quenching (Horton et al., 1991). Similar aggregation induced quenching can be also caused by chemicals having strong effects of membrane impermeable cations such as the polyamines (e.g., petruscine, spermidine, and spermine) on Chl *a* fluorescence quenching (Ioannidis and Kotzabasis, 2007). These organic compounds are synthesized by living cells, and they can quench the maximal fluorescence in the dark and stimulate NPQ in light *in vivo* in higher plants (Ioannidis and Kotzabasis, 2007; Ioannidis et al., 2012) and in algae (Ioannidis et al., 2011). This simulation of NPQ seems to be due to antenna aggregation (Tsiavos et al., 2012).

The above—mentioned phenomenon has been confirmed *in vivo*, and the effect, to a great extent, simulates proton-triggered quenching in isolated antenna (Tsiavos et al., 2012; Malliarakis et al., 2015). Further, this is in agreement with the effect of other tertiary amines—e.g., dibucaine, which has been shown to stimulate NPQ (Ruban et al., 1994; Phillip et al., 1996; Gilmore and Yamasaki, 1998) and bind to thylakoid membrane surfaces (Gilmore and Yamasaki, 1998). As Mg^2+^ accumulates on the thylakoid membrane surface when it is exposed to light (Hind et al., 1974), we can speculatethat the cations (polyamines, Mg^2+^) present on the stromal surface could synergistically stimulate qE (triggered by low lumen pH) by allowing antenna aggregation. This type of mechanism would be in line with the presence of a “*cation sensitive site”* on the stromal side of the thylakoids (Mills et al., 1976). Indeed, one from the subdomain of CP29 antenna from spinach has been proposed to be regulated by chemiosmotic factor (Ioannidis et al., 2016). However, the stimulatory effect of ions on NPQ of fluorescence is, apparently, against the proposed role of Mg^2+^ in EDL theory (see Figure 4), where Mg^2+^ induced high screening mode is observed by high Chl *a* fluorescence. It seems that there are two quite different mechanisms for cation effects on Chl *a* fluorescence yield; it could be by direct binding (no fluorescence increase, see Figure 5; possibly stimulating NPQ) or by the effect on EDL (no binding connected with fluorescence increase). However, in any case these contradictory conclusions require new experimental approaches to be used.

## General aspects of light-harvesting and ion transport

Protons have distinct effects on the efficiency of the light-harvesting process at the level of excitation energy dissipation of excess light by NPQ of excited state of Chl *a* molecules (Ruban et al., 2012; Adams et al., 2014; Demmig-Adams et al., 2014; Horton, 2014), whereas, other ions may regulate excitation distribution and redistribution, and stacking of thylakoid membranes (Barber, 1982; Staehelin and Arntzen, 1983; Minagawa, 2011; Papageorgiou and Govindjee, 2011). Proton-induced membrane stacking is a well-known phenomenon (Gerola et al., 1979; Jennings et al., 1981a,b). It is caused by neutralization of negative charges on proteins at high proton concentration (see e.g., Barber, 1980a). In photosynthetic cells, these processes are interconnected and light-induced proton pumping is intertwined with counter ion transport (Barber et al., 1974; Hind et al., 1974). Light-induced accumulation of protons in the lumen is balanced mostly by the efflux of Mg^2+^ ions (Hind et al., 1974; Chow et al., 1976; Ishijima et al., 2003) or K^+^ ions (Chow et al., 1976; Tester and Blatt, 1989; Carraretto et al., 2013) to the stroma and/or uptake of Cl^−^ ions into the lumen (Hind et al., 1974; Vambutas and Schechter, 1983; Vambutas et al., 1984, 1994). This has been confirmed by the observation of a light-induced increase in K^+^ and Mg^2+^ concentration in the stroma (Dilley and Vernon, 1965; Hind et al., 1974; Krause, 1977; Portis, 1981), and the accompanying higher Cl^−^ concentration in the lumen (Hind et al., 1974; Vambutas and Schechter, 1983).

It is not yet clear which counter ion is dominant in chloroplasts *in vivo* since most of the earlier measurements were done on isolated chloroplasts where proton exchange with the cytoplasm could be under-or over-estimated (see e.g., Hind et al., 1974). However, a different major influence of cations has been suggested (see discussion by Cruz et al., 2001) which leads to shrinking of thylakoids after illumination (Dilley and Vernon, 1965; Nobel, 1968). However, there are irreconcilable findings: thylakoid lumen shrinkage (Posselt et al., 2012), and its opposite, a swelling (Kirchhoff et al., 2011). We need an answer to this dilemma. Generally, in most experiments, Mg^2+^ has been suggested to be the major physiological counterion for H^+^ pumping into the chloroplast (Barber et al., 1974; Hind et al., 1974; Barber, 1976; Enz et al., 1993; Cruz et al., 2001; Ishijima et al., 2003) since another mobile cation, K^+^, seems to be rather bound or trapped in the chloroplast (Figure 1). Inorganic anions induce state changes in spinach thylakoid membranes (Jajoo et al., 1998) that may indicate a role for Cl^−^ as a counter ion since its transport into the thylakoid lumen could compensate for the H^+^ resulting in thylakoid membrane swelling, observed upon exposure of chloroplasts to light (Kirchhoff et al., 2011). It is also plausible that different counterions behave differently under high/low light. Indeed, counterions for H^+^ pumping are affected by lumen pH, which would indicate that different ions act as counter-ions at low light (Cl^−^) and at high light [Mg^2+^] (Ben-Hayyim, 1978).

All the available data support the concept that the role of protons and of all other ions in the regulation of photosynthesis is interconnected since proton/cation antiport (proton/anion symport) is required during photophosphorylation as the electrochemical part of pmf (Ort and Melandri, 1982; Cruz et al., 2001) is reduced in the presence of light in higher plant chloroplasts (Figure 1). This idea is further supported by the fact that stacking of thylakoid membranes can be induced either by high proton or with high Mg^2+^ concentration (cf. Gerola et al., 1979; Jennings et al., 1979, 1981a,b; Barber, 1980a). Thus, research on the regulation of light-harvesting efficiency requires a new approach when the effects, and mechanisms, of both protons and all other ions will be addressed simultaneously. This is even more important in light of the discovery of several ion channels and transporters in the chloroplast (see, e.g., Carraretto et al., 2013; Armbruster et al., 2014; Hamamoto and Uozumi, 2014; Kunz et al., 2014; Herdean et al., 2016; also see recent reviews by Checchetto et al., 2013; Hanikenne et al., 2014; Pfeil et al., 2014; Finazzi et al., 2015; Xu et al., 2015; Carraretto et al., 2016).

## Author contributions

Both the authors have made substantial, direct and intellectual contribution to the work, and approved it for publication.

### Conflict of interest statement

The authors declare that the research was conducted in the absence of any commercial or financial relationships that could be construed as a potential conflict of interest.

## Dedication

Dedicated to the pioneering research of James (Jim) Barber on many aspects of photosynthesis including the electrostatic properties of the chloroplast thylakoid membrane, and who received the 2016 Communication Award of the International Society of Photosynthesis Research (ISPR).

## Abbreviations

Chl: chlorophyll
DCMU, 3-(3, 4-dichlorophenyl)-1: 1-dimethylurea
EDL: electrical double layer
PS I (II): photosystems I (II)
TM: thylakoid membrane
